# Distortion of K_B_ estimates of endothelin‐1 ET_A_ and ET_B_ receptor antagonists in pulmonary arteries: Possible role of an endothelin‐1 clearance mechanism

**DOI:** 10.1002/prp2.374

**Published:** 2017-12-05

**Authors:** James A. Angus, Richard J. A. Hughes, Christine E. Wright

**Affiliations:** ^1^ Cardiovascular Therapeutics Unit Department of Pharmacology and Therapeutics University of Melbourne Melbourne Vic. Australia

**Keywords:** ambrisentan, bosentan, BQ788, endothelin‐1, ET_A_ receptors, ET_B_ receptor‐mediated clearance mechanism, ET_B_ receptors, macitentan, sarafotoxin S6c

## Abstract

Dual endothelin ET_A_ and ET_B_ receptor antagonists are approved therapy for pulmonary artery hypertension (PAH). We hypothesized that ET_B_ receptor‐mediated clearance of endothelin‐1 at specific vascular sites may compromise this targeted therapy. Concentration‐response curves (CRC) to endothelin‐1 or the ET_B_ agonist sarafotoxin S6c were constructed, with endothelin receptor antagonists, in various rat and mouse isolated arteries using wire myography or in rat isolated trachea. In rat small mesenteric arteries, bosentan displaced endothelin‐1 CRC competitively indicative of ET_A_ receptor antagonism. In rat small pulmonary arteries, bosentan 10 μmol L^−1^ left‐shifted the endothelin‐1 CRC, demonstrating potentiation consistent with antagonism of an ET_B_ receptor‐mediated endothelin‐1 clearance mechanism. Removal of endothelium or L‐NAME did not alter the EC_50_ or Emax of endothelin‐1 nor increase the antagonism by BQ788. In the presence of BQ788 and L‐NAME, bosentan displayed ET_A_ receptor antagonism. In rat trachea (ET_B_), bosentan was a competitive ET_B_ antagonist against endothelin‐1 or sarafotoxin S6c. Modeling showed the importance of dual receptor antagonism where the potency ratio of ET_A_ to ET_B_ antagonism is close to unity. In conclusion, the rat pulmonary artery is an example of a special vascular bed where the resistance to antagonism of endothelin‐1 constriction by ET dual antagonists, such as bosentan or the ET_B_ antagonist BQ788, is possibly due to the competition of potentiation of endothelin‐1 by blockade of ET_B_‐mediated endothelin‐1 clearance located on smooth muscle and antagonism of ET_A_‐ and ET_B_‐mediated contraction. This conclusion may have direct application for the efficacy of endothelin‐1 antagonists for treating PAH.

**Abbreviations**
D_100_: artery (mesenteric or tail) internal diameter (µm) at an equivalent transmural pressure of 100 mm Hg
D_20_: artery (pulmonary) internal diameter (µm) at an equivalent transmural pressure of 20 mm Hg
Emax: maximum possible effect for the agonist
KPSS: isotonic potassium physiological salt solution (K^+^ 124 mmol L^−1^ for arteries or K^+^ 62 mmol L^−1^ for trachea)
*p*EC_50_: negative log_10_ M concentration of agonist that evokes the half‐maximal response
*p*K_B_: negative log_10_ M concentration of antagonist that shifted the agonist endothelin‐1 EC_50_ twofold to the right
PSS: physiological salt solution

## 1 INTRODUCTION

In rats,^1^ rabbits,^2^ and nonhuman primates,^3^ dual ET_A_ and ET_B_ receptor antagonists or ET_B_‐selective endothelin‐1 antagonists increased the immunoreactive endothelin‐1 plasma level acutely by 3‐ to 10‐fold. After chronic oral dosing in rats with A‐182086, a dual ET_A_ and ET_B_ antagonist, the endothelin‐1 plasma levels rose by more than 24‐fold after 9 days.^4^ Micro positron emission tomography using ^18^F‐labeled endothelin‐1 in anesthetized rats confirmed that endothelin‐1 rapidly binds to rat lung and is cleared from the circulation (*t*_0.5_ 0.43 minutes).^5^ Pretreatment with the ET_B_‐selective antagonist BQ788 decreased the endothelin‐1 clearance by 85%.

**Figure 1:**
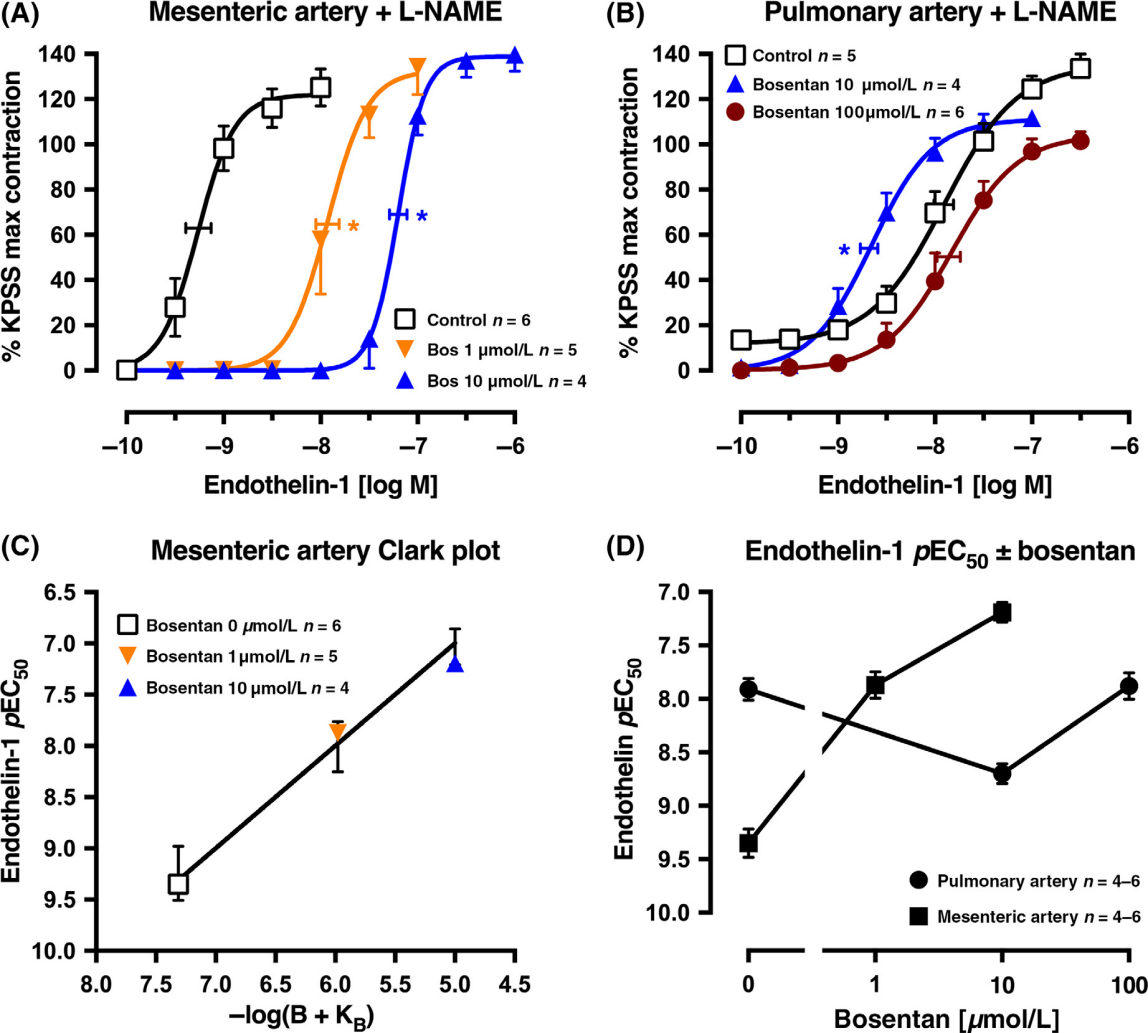
Average single exposure concentration‐contraction curves to endothelin‐1 in rat (A) mesenteric artery (n* *=* *15) and (B) pulmonary artery (n* *=* *15), pretreated with L‐NAME 100 μmol L^−1^, in the absence Control, (0 μmol L^−1^) or presence of bosentan 1, 10 or 100 μmol L^−1^. Data are expressed as % KPSS maximum contraction (y axis). (C) Clark plot display for the relationship in the rat mesenteric artery between the endothelin‐1 *p*EC_50_ values (y axis; −log M) and −log(B + K_B_) where B is concentration of bosentan (0, 1, or 10 μmol L^−1^) and K_B_ is the global‐fitted dissociation constant. The error bars are ± 2 SEM of the difference between the nonlinear regression‐fitted *p*EC_50_ values for endothelin‐1 and the *p*EC_50_ values fitted for the individual artery for each concentration of bosentan (B). (D) The *p*EC_50_ values for the endothelin‐1 curves in (A) and (B) are plotted on the y axis against the bosentan concentration (x axis) for each artery type. Vertical error bars in (A, B, and D) are ± 1 SEM (those not shown are contained within the symbol). Horizontal error bars (A‐B) represent the EC_50_ ± 1 SEM. n, number of arteries isolated from separate animals. *P <* *.05, *p*EC_50_ values compared with respective control (0 μmol L^−1^) *p*EC_50_ values. Variations in n are due to violation of predetermined criteria: mesenteric arteries that contracted to KPSS with <3 mN force or pulmonary arteries that contracted to KPSS with <1 mN force

While this intriguing mechanism of endothelin‐1 clearance by ET_B_ receptors was first determined in vivo, we asked, could this mechanism affect the pharmacodynamics of endothelin‐1 interactions with ET_A_ and ET_B_ receptors mediating smooth muscle contraction in isolated tissue assays when determining the *p*K_B_ of endothelin‐1 receptor antagonists? The impact of sites of loss of agonist or antagonist concentrations on *p*K_B_ estimations has been observed in the acid‐secreting mouse stomach (figure 1 in Angus and Black^6^) and further developed by Kenakin.^7^ Indeed, we have previously reported that endothelin‐1 concentration‐contraction curves in rat small interlobar pulmonary arteries were surprisingly LEFT‐shifted; ie, endothelin‐1 contractions were “potentiated” in the presence of the dual ET_A_ and ET_B_ antagonist bosentan 10 μmol L^−1^,^8^ an observation that is consistent with blockade of a site of loss of endothelin‐1.

Here, we report the pharmacodynamic interactions and analyses of endothelin‐1 receptor antagonists in a range of isolated arteries and tracheal smooth muscle preparations with endothelin‐1 and the selective ET_B_ receptor agonist venom peptide sarafotoxin S6c. Some arteries were treated with L‐NAME or had the endothelial cell layer removed. Our results show that the localized ET_B_ clearance mechanism for endothelin‐1 on smooth muscle cells could explain the dramatic effect on the estimation of the dissociation constant for ET_A_ and ET_B_ antagonists when endothelin‐1 is used as the agonist and the endothelin‐1 clearance mechanism is present.

The conclusions provide a theoretical framework to test for the “ideal” dual ET_A_ and ET_B_ receptor antagonist if significant antagonism is to occur at ET_A_ or ET_B_ constrictor receptors and the ET_B_ receptor‐mediated clearance of endothelin‐1 is blocked which potentiates the potency of endothelin‐1. This clearance mechanism, thus, joins other well‐known mechanisms of ET_B_‐mediated endothelin‐1 release of thromboxane A_2_, prostacyclin, and nitric oxide that would either enhance or functionally antagonize ET_A_‐ or ET_B_‐mediated vasoconstriction.^9^, ^10^, ^11^, ^12^

## 2 MATERIALS AND METHODS

The ethics committee of the University of Melbourne approved the experiments in accordance with the *Australian Code for the Care and Use of Animals for Scientific Purposes* (8^th^ edition, 2013; National Health & Medical Research Council, Canberra, Australia). Animal studies are reported in compliance with the ARRIVE guidelines.^13^, ^14^ Male Sprague‐Dawley rats (280‐320 g; Biomedical Sciences Animal Facility, University of Melbourne, Australia) and male Swiss mice (30‐40 g; Animal Resources Centre, Murdoch, WA, Australia) were used in this study. Animals were housed (3‐4 per high‐topped cage with shredded paper bedding) at 22°C on a 12‐hour light/dark cycle with access to food and water ad libitum. Rats and mice were individually placed in a secure chamber and deeply anesthetized by inhalation of 5% isoflurane in oxygen, then killed by rapid excision of the heart. The rat and mouse tissues were rapidly excised and placed in cold physiological salt solution (PSS) with the following composition (mM): NaCI 119; KCl 4.69; MgSO_4_.7H_2_O 1.17; KH_2_PO_4_ 1.18; glucose 5.5; NaHCO_3_ 25; CaCl_2_.6H_2_O 2.5; EDTA 0.026 and saturated with carbogen (O_2_ 95%; CO_2_ 5%) at pH 7.4. Tissues were pinned down on a Silastic‐covered petri dish filled with cold PSS. A minimum of 5 rats or mice was used for each experimental group, with exact *n* values shown in the figure legends or Results section. Group sizes were equal by design; however, variations due to predetermined criteria (described in the methodology) are explained in the figure legends. Animal tissues were randomized to treatment groups.

### 2.1 Arteries

As previously described,^15^ third‐order rat and mouse mesenteric arteries, rat and mouse pulmonary arteries, and mouse tail arteries were dissected clear of their connective tissue and prepared as 2‐mm‐long segments for mounting on 40‐μm diameter wires for isometric force measurement in Mulvany and Halpern style myographs (model 620M, Danish Myo Technology, Aarhus, Denmark). Responses were captured by a Powerlab 4/30 A/D converter (ADInstruments, Sydney, Australia) and measured on a computer running LabChart 7 data acquisition software (ADInstruments).

After 30‐minute equilibration in PSS at 37°C, the arteries were passively stretched under micrometer (Mitutoyo, Kawasaki, Japan) control according to the normalization protocol to determine the internal diameter at equivalent transmural pressure of 100 mm Hg (D_100_) for all arteries, except for the pulmonary artery where 20 mm Hg was used (D_20_). The micrometer was then adjusted to decrease the passive stretch to an equivalent diameter of 90% of D_100_ (or 90% of D_20_, as applicable) and the artery remained at that setting of passive stretch for the remainder of the experiment.^15^, ^16^ Thirty minutes later the arteries were exposed for 2 minutes to potassium depolarizing solution (K^+^ replacing Na^+^ in PSS, ie, 124 mmol L^−1^; termed KPSS), before replacing with PSS. Subsequent responses were expressed as a % of this KPSS reference contraction in each artery. Rat or mouse mesenteric arteries that contracted to KPSS with <3 mN force, mouse tail arteries that contracted to KPSS with <20 mN force, or rat and mouse pulmonary arteries that contracted to KPSS with <1 mN force were considered as violations of predetermined criteria. As a further test of viability of the artery, a single 2‐minute exposure to 10 μmol L^−1^ noradrenaline was performed and then replaced with drug‐free PSS. To test the integrity of the endothelium, arteries were precontracted with noradrenaline 1 μmol L^−1^ (which contracts to about 80% of KPSS) and acetylcholine 1 μmol L^−1^ was added which would normally completely relax the artery in <30 seconds to the baseline force. Some arteries were equilibrated for 30 minutes with L‐NAME (N_ω_‐nitro‐L‐arginine methyl ester; 100 μmol L^−1^) and one concentration of an endothelin antagonist (bosentan 1, 10, or 100 μmol L^−1^; BQ788 0.3, 1, or 3 μmol L^−1^). In one study, BQ788 3 μmol L^−1^ and 0, 1, or 10 μmol L^−1^ bosentan were equilibrated before the concentration‐response curve was constructed to endothelin‐1. In each study, the artery was then exposed to a single cumulative concentration‐contraction curve (0.1 nmol L^−1^ to 3 μmol L^−1^, depending on agonist, tissue, and treatment) to either sarafotoxin S6c or endothelin‐1, added in half‐log_10_ M increments allowing time for the contraction to reach a plateau before raising the concentration.

### 2.2 Endothelium removal

In rat small pulmonary artery, the normalization procedure was completed before testing the relaxation to acetylcholine 1 μmol L^−1^ in arteries contracted by U46619 (0.1 μmol L^−1^). The artery passive force was then relaxed, and a human black hair was inserted into the artery lumen. Lateral movement of the hair and careful rotation of the artery loosely suspended on the 2 wires removed the endothelial cells. The passive force was reapplied to the level prior to the endothelial cell removal and the acetylcholine (1 μmol L^−1^) test repeated in the presence of U46619 (0.1 μmol L^−1^). Failure to relax to acetylcholine was considered the functional test of endothelial cell removal. The endothelium‐denuded arteries can still deliver a full relaxation response to sodium nitroprusside 1 μmol L^−1^.

### 2.3 Trachea

The main trachea (10 mm long) was dissected free from the rat, cut into 2‐ to 3‐mm‐long ring segments, and mounted on wires in 15‐mL organ baths (see figure 1 in Angus and Wright),^15^ used for large diameter ring segments. In some trachea ring segments, the epithelial cell layer was removed by using a splinter of wood and gently rubbing the lumen for 1 minute. The rings were stretched to a passive force of 1 g and equilibrated in PSS at 37°C for 60 minutes. A reference contraction to KPSS (62 mmol L^−1^ for trachea, see Clozel et al^17^) was obtained before washing the tissue with drug‐free PSS. Subsequent responses were expressed as a % of this KPSS reference contraction in each tracheal ring. Tracheae that contracted to KPSS with <1 g force were considered as violations of predetermined criteria. The resting force was readjusted to 1 g and the trachea left to equilibrate for 30 minutes in the absence or presence of bosentan (3, 10, or 30 μmol L^−1^). A single concentration‐contraction curve to sarafotoxin S6c or endothelin‐1 was constructed up to a maximum concentration of 0.3 μmol L^−1^ for sarafotoxin S6c or 3 μmol L^−1^ for endothelin‐1.

### 2.4 Drugs

Drugs used were acetylcholine bromide (Sigma, St Louis, MO, USA); ambrisentan (Selleckchem, Houston, TX, USA); bosentan sodium salt (Selleckchem); BQ788 sodium salt (Peptides International, Louisville, KY, USA); endothelin‐1 (Genscript, Piscataway, NJ, USA); macitentan (Selleckchem); N_ω_‐nitro‐L‐arginine methyl ester hydrochloride (Sigma); (‐)‐noradrenaline bitartrate (Sigma); and sarafotoxin S6c (Auspep, Parkville, Victoria, Australia). All drugs were dissolved in MilliQ water except for endothelin‐1 which was dissolved in 10% dimethylformamide to 10^−4^ mol L^−1^, then diluted in MilliQ water, macitentan which was dissolved in DMSO to 10^−3^ mol L^−1^, then diluted in MilliQ water, and BQ788 which was dissolved in DMSO to 10^−4^ mol L^−1^.

### 2.5 Statistics and analyses

All data are expressed as mean ± SEM from *n* experiments. The data and analyses comply with the recommendations on experimental design and analysis in pharmacology.^18^ All contraction responses to endothelin‐1 or sarafotoxin S6c were measured as a % of the Emax (maximum response to agonist) to KPSS within each artery or tracheal ring. Each individual sigmoidal concentration‐contraction curve to endothelin‐1 or sarafotoxin S6c in the absence or presence of an endothelin receptor antagonist was fitted using Prism 7 (GraphPad Software, La Jolla, CA, USA). The *p*EC_50_ ± SEM values (−log_10_ M EC_50_) were determined for each treatment group. In endothelin‐1 experiments in rat trachea, the concentration‐contraction curves were not fitted as Emax values were not defined; instead, endothelin‐1 *p*EC_50_ values were calculated at responses of 50% KPSS maximum contraction. *p*EC_50_ values in treatment groups were compared to the respective control group with a one‐way ANOVA and Dunnett's post hoc test (Prism 7). Blinding was not performed for this study as all experiments yielded strict quantitative data.

### 2.6 Clark plot and analyses

Endothelin‐1 rapidly activates the respective ET_A_ or ET_B_ receptors before being internalized for recycling (ET_A_) or destruction (ET_B_) (see Bremnes et al^19^ and Paasche et al^19^, ^20^). This phenomenon makes it particularly difficult to establish multiple concentration‐response curves within a particular artery. In practical terms, the ET_A_ or ET_B_ receptor may be rapidly activated, but the resultant calcium mobilization and contraction takes a considerable time to develop even in small arteries <200 μm diameter. Thus, we routinely designed our experiments around a single cumulative concentration‐response curve in the presence or absence of an antagonist concentration.

Our chosen experimental design of only one concentration‐response curve per tissue does not allow for Schild plot analyses or determination of concentration ratios within tissue. By preference, we used the Clark plot and global fit analysis with its robust advantages.^21^ To determine the antagonist dissociation constant (K_B_) for each endothelin antagonist, we applied the global regression method^22^ that was simplified from that developed originally by Stone and Angus.^21^ A computer‐based nonlinear regression was performed to solve for K_B_ (*p*K_B_ = −log K_B_) by iterative approximation for ALL the endothelin‐1 (or sarafotoxin S6c) *p*EC_50_ values in the absence or presence of antagonist (B) concentrations thus:

(1) $$p{\text{EC}}_{50}=-\text{log}\left[{\left(B\right)}^{n}+10^{-pK}B\right]-\text{log}c$$

where n is a “power departure” equivalent to allowing the slope of a Schild plot to vary from unity (see Lew and Angus^22^).

Having solved *p*K_B_, the relationship between the mean *p*EC_50_ values of the actual data were plotted against the antagonist concentration −log(B  +  K_B_) at concentrations of bosentan (0, 1, 3, 10, 30, or 100 μmol L^−1^), ambrisentan (0, 1, 3, or 10 μmol L^−1^), or macitentan (0, 0.3, 1, or 10 μmol L^−1^). This graphical display was named the Clark plot by Stone and Angus^21^ as it was similar to the plot developed by Clark^23^ of log(agonist) vs log(antagonist) at equal level of response. There are 2 important ways to test whether the concentration‐response curves are displaced to the right of the control *p*EC_50_ according to simple competitive antagonism. First, whether the 95% confidence limits for n contains 1; if so, the equation (1) is fitted where n = 1. Second, the error bars on the Clark plot are ± 2 times the standard error of the differences between the observed endothelin‐1 (or sarafotoxin S6c) *p*EC_50_ values and the predicted *p*EC_50_ values from the fitted equation (1). This provides an estimate of the confidence band around the line. If the point showing the average of the observed *p*EC_50_ values at a level of −log(B  +  K_B_) fell outside the error bar, this would indicate a departure from the simple competitivity model.

For comparison of *p*K_B_ values between each antagonist in different settings, an unpaired Student's *t* test (Prism 7) was performed. Statistical significance was taken when *P *<* *.05.

## 3 RESULTS

### 3.1 Rat mesenteric and pulmonary small arteries

In rat small mesenteric arteries (i.d. 352 ± 6 μm), single endothelin‐1 concentration‐response curves had a *p*EC_50_ of 8.12 ± 0.02 and an Emax of 108 ± 5% KPSS (n* *=* *4; data not shown). In the presence of L‐NAME (100 μmol L^−1^), the *p*EC_50_ for endothelin‐1 was 9.35 ± 0.13 (n* *=* *6), significantly higher (17‐fold more potent) than in the absence of L‐NAME, and the Emax was 123 ± 9% KPSS (Figure 1A). In rat small second‐order pulmonary arteries (524 ± 20 μm i.d.), the *p*EC_50_ for endothelin‐1 was 7.55 ± 0.20 with an Emax of 124 ± 4% KPSS (n* *=* *5; data not shown). In the presence of L‐NAME (100 μmol L^−1^), the *p*EC_50_ was 7.91 ± 0.10, not significantly different from control, and the Emax was 135 ± 7% KPSS (n* *=* *5; Figure 1B). In the presence of L‐NAME and bosentan 1 and 10 μmol L^−1^, the endothelin‐1 concentration‐response curves were right‐shifted in a competitive manner in the rat mesenteric artery (Figure 1A), but significantly left‐shifted with bosentan 10 μmol L^−1^ in the rat pulmonary artery (Figure 1B). In the presence of bosentan 100 μmol L^−1^, the endothelin‐1 curve was located not significantly different to the control in the presence of L‐NAME (Figure 1B). For the mesenteric artery, the Clark plot and analyses indicate a *p*K_B_ of 7.31 ± 0.16 (n* *=* *15 points), congruent with the model of competitive antagonism (Figure 1C). A display of the *p*EC_50_ values for endothelin‐1 concentration‐response curves shows the significantly different control *p*EC_50_ for endothelin‐1 in the 2 artery types and the opposite effect on the *p*EC_50_ by bosentan 10 μmol L^−1^, all in the presence of L‐NAME (Figure 1D). Clearly, the presence of 100 μmol L^−1^ bosentan had very little effect in antagonizing the endothelin‐1 contraction when compared with control (0 μmol L^−1^ bosentan) in the pulmonary artery.

**Figure 2:**
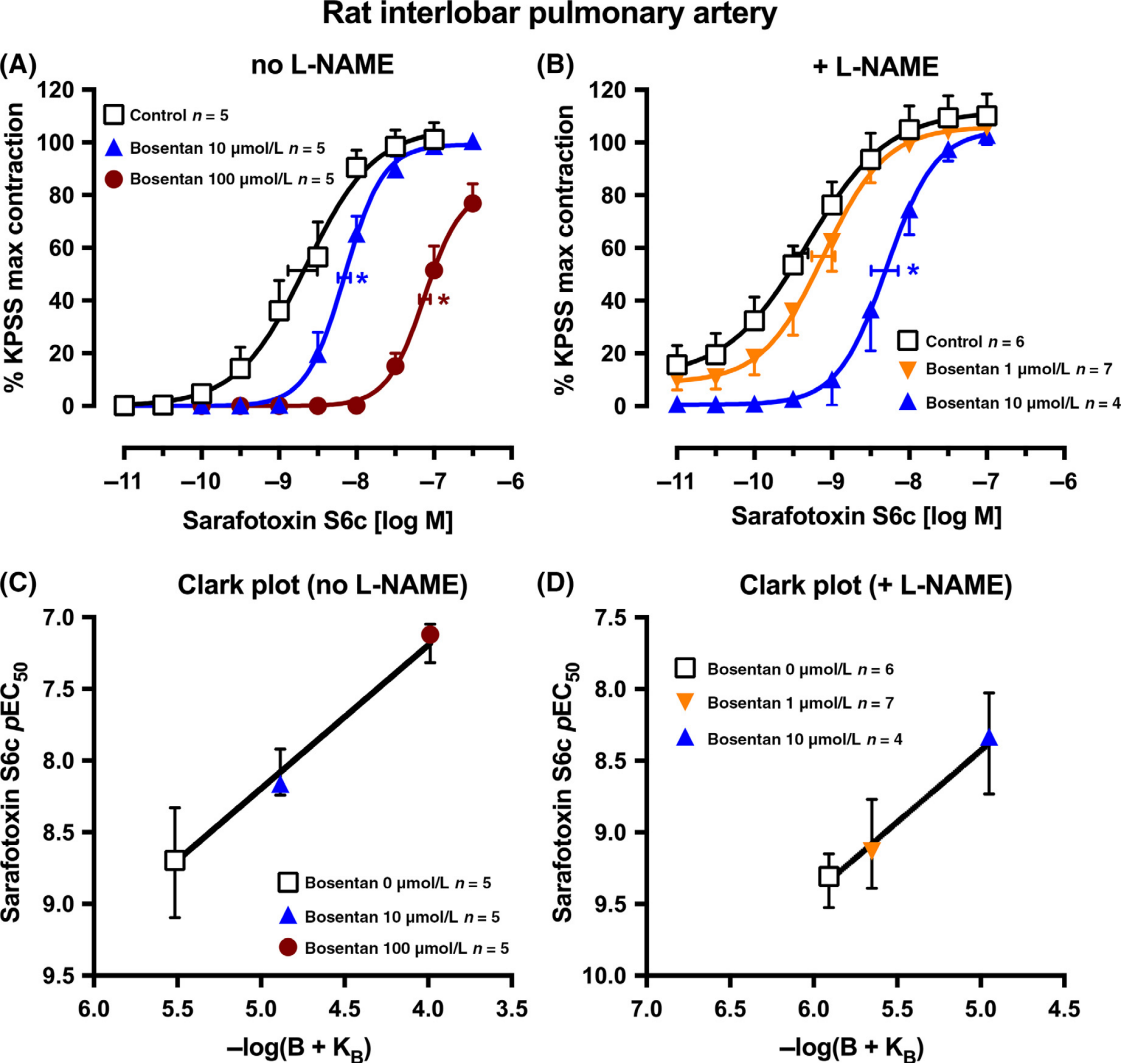
Average single exposure concentration‐contraction curves to sarafotoxin S6c in rat pulmonary artery in the (A) absence or (B) presence of L‐NAME 100 μmol L^−1^ and of bosentan 0 (Control), 1, 10, or 100 μmol L^−1^. Data are expressed as % KPSS maximum contraction (y axis). Vertical error bars are ± 1 SEM (those not shown are contained within the symbol). Horizontal error bars represent the EC_50_ ± 1 SEM. (C‐D) Clark plot displays for the corresponding figure panel above for the relationship between the sarafotoxin S6c *p*EC_50_ values (y axis; −log M) and −log(B + K_B_) values (see legend for Figure 1C) in the absence (C) or presence (D) of L‐NAME. n, number of arteries isolated from separate animals. *P <* *.05, *p*EC_50_ values compared with respective control (0 μmol L^−1^) *p*EC_50_ values. Variations in *n* are due to violation of a predetermined criterion: arteries that contracted to KPSS with <1 mN force

The failure to obtain an estimate of the *p*K_B_ for bosentan in rat small pulmonary artery, possibly due to the removal of the agonist endothelin‐1, prompted the use of the nonendogenous ET_B_‐selective ligand sarafotoxin S6c in the absence or presence of L‐NAME. The control (0 μmol L^−1^ bosentan) curve was more potent (4.1‐fold) in the presence of L‐NAME (*p*EC_50_ with L‐NAME 9.31 ± 0.09, n* *=* *6, and without L‐NAME 8.70 ± 0.19, n* *=* *5; Figure 2A,B), suggesting a small effect of NO release in functionally antagonizing the contraction. The Clark plots and analyses show that bosentan is a competitive antagonist with similar *p*K_B_ values in the absence or presence of L‐NAME of 5.52 ± 0.17 (n* *=* *15 points) and 5.91 ± 0.24 (n* *=* *17 points), respectively (Figure 2C,D).

**Figure 3:**
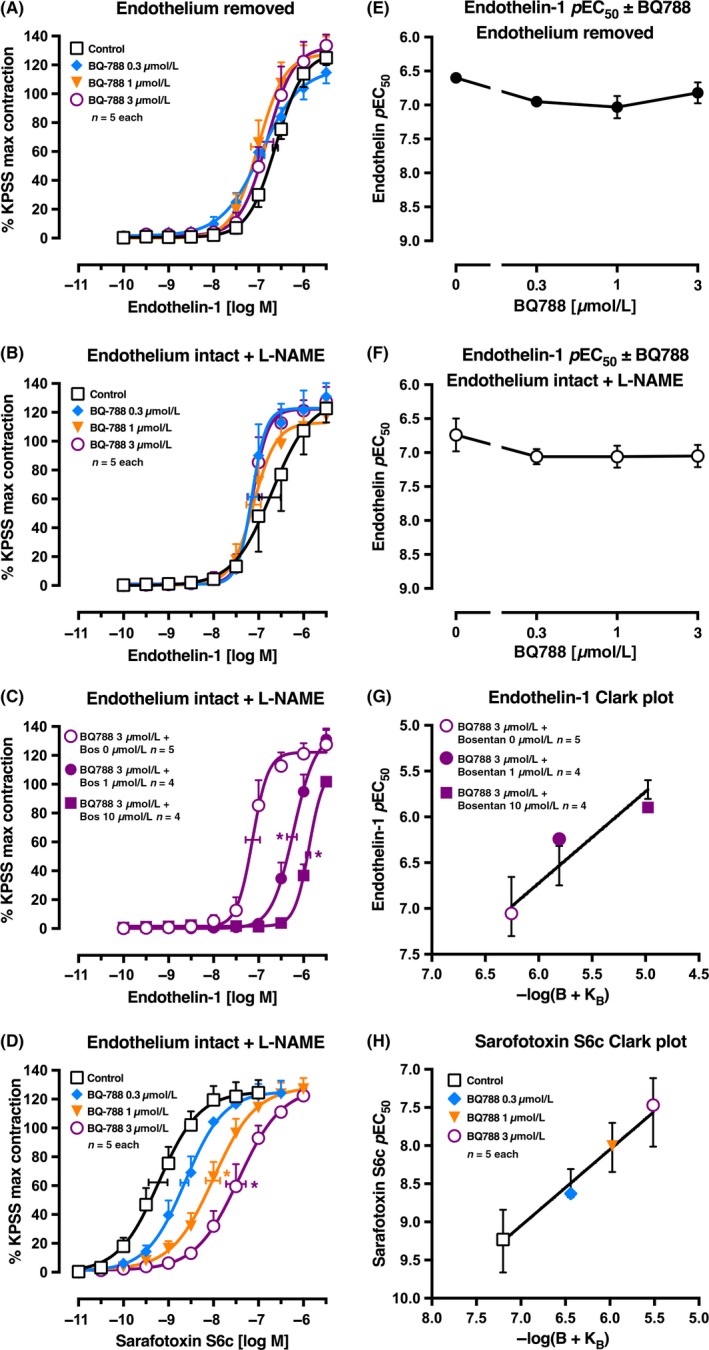
Average single exposure concentration‐contraction curves to (A‐C) endothelin‐1 or (D) sarafotoxin S6c in rat pulmonary artery in the (A) absence of endothelium or (B‐D) presence of endothelium plus L‐NAME 100 μmol L^−1^. (A, B, and D) Curves were completed in the absence (Control, 0 μmol L^−1^) or presence of BQ788 0.3, 1, or 3 μmol L^−1^. (C) Curves were completed in the presence of BQ788 3 μmol L^−1^ plus bosentan 0, 1, or 10 μmol L^−1^. Data are expressed as % KPSS maximum contraction (y axis). (E‐F) The *p*EC_50_ values from (A‐B) are plotted on the y axis against the BQ788 concentration (x axis). Vertical error bars (A‐F) are ± 1 SEM (those not shown are contained within the symbol). Horizontal error bars (A‐D) represent the EC_50_ ± 1 SEM. (G‐H) Clark plot displays for the corresponding left figure panel for the relationship between the (G) endothelin‐1 or (H) sarafotoxin S6c *p*EC_50_ values (y axis; −log M) and −log(B + K_B_) values (see legend for Figure 1C) in the presence of L‐NAME. *P <* *.05, *p*EC_50_ values compared with respective control (0 μmol L^−1^) *p*EC_50_ values. *n*, number of arteries isolated from separate animals. Variations in *n* are due to violation of a predetermined criterion: arteries that contracted to KPSS with <1 mN force

The more direct test for the role of ET_B_ receptors in the endothelial clearance of endothelin‐1 was concluded from the interaction of the highly selective ET_B_ receptor antagonist BQ788 and endothelin‐1 in the absence of the endothelium (Figure 3A). Again BQ788 (0.3‐3 μmol L^−1^) appeared to slightly left‐shift (potentiate) the endothelin‐1 concentration‐response curve *p*EC_50_. Moreover, this family of curves was similar in pattern to the curves in the presence of L‐NAME and endothelium (Figure 3B,E‐F), suggesting that ET_B_‐mediated clearance of endothelin‐1 may well be dependent on the smooth muscle cells in this artery rather than on the endothelium. To calibrate the ET_B_ receptor on smooth muscle in the absence of NO release or clearance, we tested individual concentration‐response curves for sarafotoxin S6c with increasing concentrations of BQ788 in the presence of L‐NAME (100 μmol L^−1^) (Figure 3D). The global fit and Clark plot gave a *p*K_B_ of 7.20 ± 0.21 (n* *=* *20 points; Figure 3H). In the presence of BQ788 3 μmol L^−1^ to antagonize the ET_B_‐mediated clearance of endothelin‐1 and antagonize the ET_B_‐mediated contraction, endothelin‐1 still contracted the pulmonary artery with a *p*EC_50_ of 7.05 ± 0.16 indicating that ET_A_ receptors were now operating (Figure 3C). To test this, equilibration with bosentan 0, 1, and 10 μmol L^−1^ gave right‐shifted concentration‐response curves and a *p*K_B_ of 6.26 ± 0.23 (n* *=* *13 points; Clark plot, Figure 3G). Note that the Clark plot display indicates that competitivity was not achieved as points lie outside the error bars for bosentan 1 and 10 μmol L^−1^.

Evidence that L‐NAME or endothelial cell removal had abolished the relaxation to acetylcholine 1 μmol L^−1^ was shown by the result that before treatment with L‐NAME or endothelial removal the relaxation to acetylcholine 1 μmol L^−1^ as a % of the precontractile tone was −54 ± 4% (n* *=* *19) or −56 ± 2% (n* *=* *18), respectively, and after treatment was −2 ± 2% or −1 ± 2%, respectively (data not shown).

### 3.2 Other arteries

**Figure 4:**
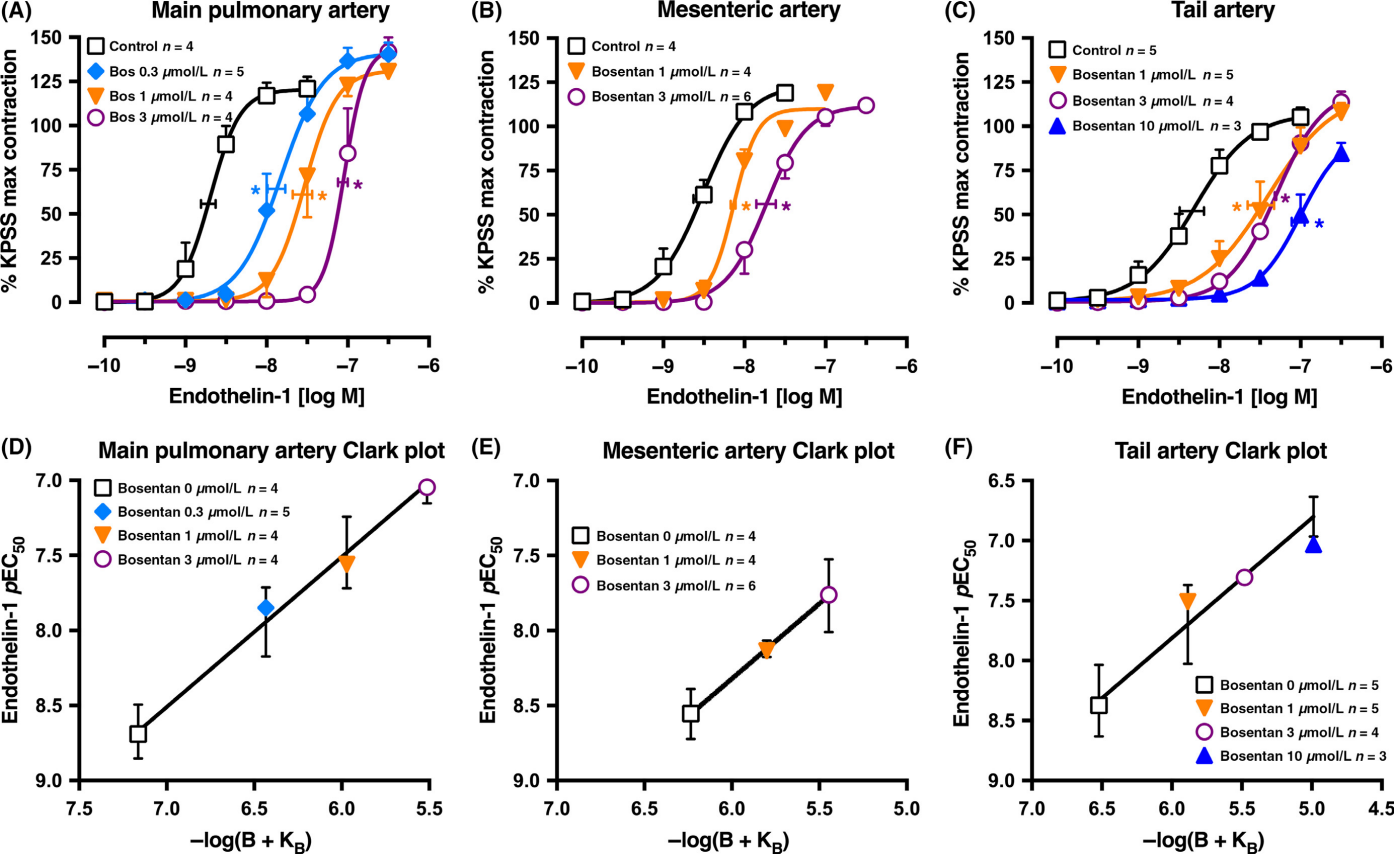
Average single exposure concentration‐contraction curves to endothelin‐1 in Swiss mouse isolated (A) main pulmonary, (B) mesenteric, and (C) tail arteries in the absence and presence of bosentan (0 (Control), 0.3, 1, 3, or 10 μmol L^−1^). Data are expressed as % KPSS maximum contraction (y axis). Vertical error bars are ± 1 SEM (those not shown are contained within the symbol). Horizontal error bars represent the EC_50_ ± 1 SEM. (D‐F) Clark plot displays for the corresponding figure panel above for the relationship between the endothelin‐1 *p*EC_50_ values (y axis; −log M) and −log(B + K_B_) values (see legend for Figure 1C). *n*, number of arteries isolated from separate animals. *P <* *.05, *p*EC_50_ values compared with respective control (0 μmol L^−1^) *p*EC_50_ values. Variations in *n* are due to violation of predetermined criteria: pulmonary arteries that contracted to KPSS with <1 mN force; mesenteric arteries that contracted to KPSS with <3 mN force; or tail arteries that contracted to KPSS with <20 mN force

In the mouse, we examined 3 different arteries to determine if the responses to bosentan and endothelin‐1 in the small pulmonary artery of the rat could be replicated. In the main pulmonary artery (i.d. 648 ± 20 μm), mesenteric artery (i.d. 275 ± 15 μm), and tail artery (i.d. 370 ± 6 μm), the patterns of endothelin‐1 concentration‐response curves and antagonism by bosentan were similar (Figure 4A‐C). The Clark plots and analyses showed *p*K_B_ values for bosentan of 7.16 ± 0.13 (n* *=* *17 points), 6.24 ± 0.16 (n* *=* *14 points), and 6.52 ± 0.18 (n* *=* *17 points) in the pulmonary, mesenteric, and tail arteries, respectively, and complied with the model of simple competitivity (Figure 4D‐F).

### 3.3 Macitentan

**Figure 5:**
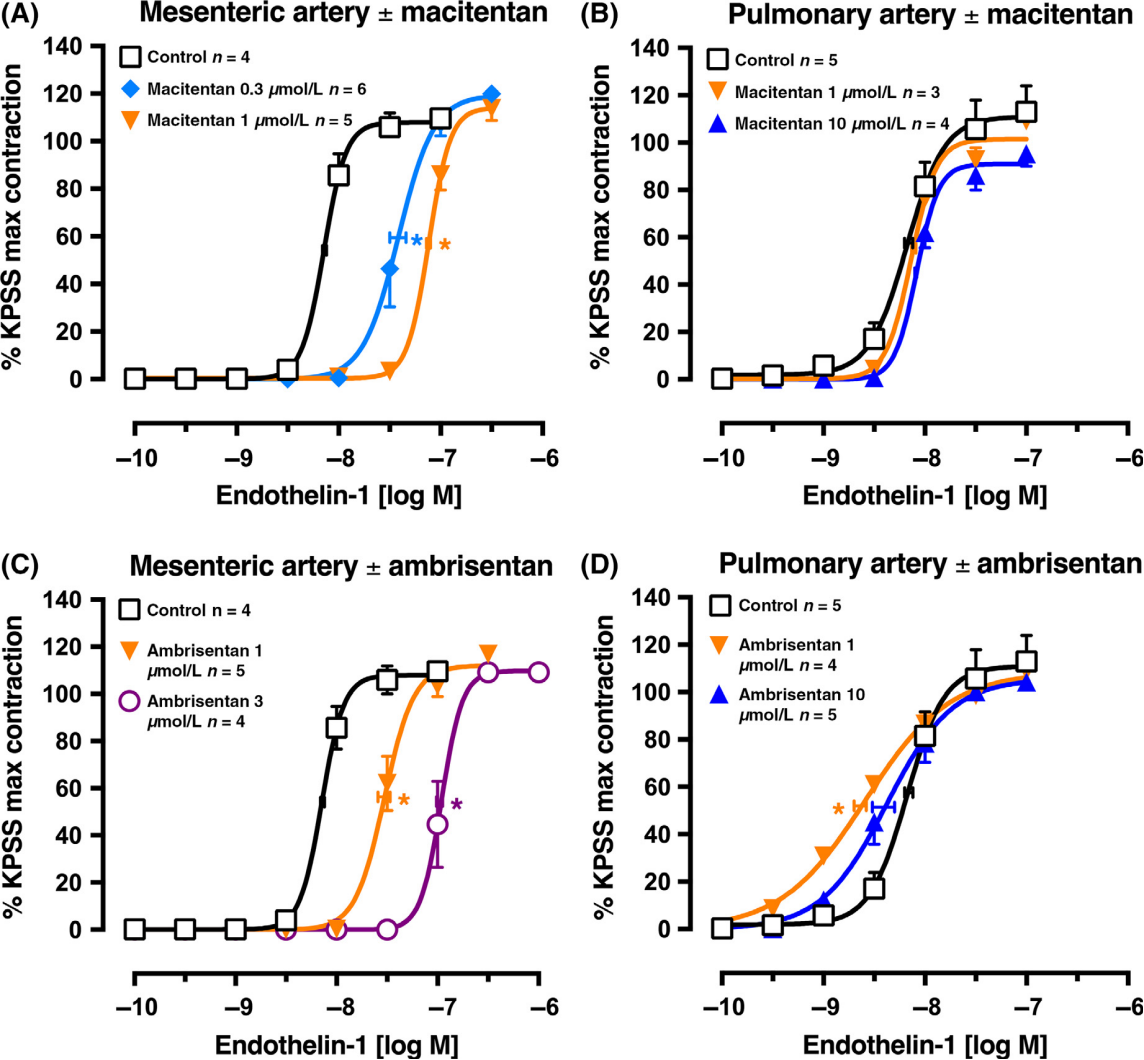
Average single exposure concentration‐contraction curves to endothelin‐1 in rat (A) and (C) mesenteric artery (n* *=* *13‐15) or (B) and (D) pulmonary artery (n* *=* *12‐14) in the absence (Control, 0 μmol L^−1^) or presence of (A‐B) macitentan 0.3, 1, or 10 μmol L^−1^ or (C‐D) ambrisentan 1, 3, or 10 μmol L^−1^. Data are expressed as % KPSS maximum contraction (y axis). Vertical error bars are ± 1 SEM (those not shown are contained within the symbol). Horizontal error bars represent the EC_50_ ± 1 SEM. *P <* *.05, *p*EC_50_ values compared with respective control (0 μmol L^−1^) *p*EC_50_ values. Variations in *n* are due to violation of predetermined criteria: mesenteric arteries that contracted to KPSS with <3 mN force or pulmonary arteries that contracted to KPSS with <1 mN force

In the rat small mesenteric artery, macitentan (0.3 and 1 μmol L^−1^) was a potent competitive endothelin‐1 receptor antagonist (Figure 5A). The Clark plot and analyses gave a *p*K_B_ of 7.05 ± 0.10 (n* *=* *15 points) and fitted the competitive model. The endothelin‐1 concentration‐contraction curves in the rat small pulmonary artery were completely unaffected by 1 and 10 μmol L^−1^ macitentan, as shown in Figure 5B.

### 3.4 Ambrisentan

In the rat small mesenteric artery, ambrisentan (1 and 3 μmol L^−1^) was a potent competitive endothelin‐1 receptor antagonist (Figure 5C). The Clark plot and analyses gave a *p*K_B_ of 6.60 ± 0.07 (n* *=* *13 points) and fitted the competitive model. In contrast, endothelin‐1 concentration‐contraction curves in the rat small pulmonary artery were slightly left‐shifted from control by ambrisentan 1 μmol L^−1^ before showing a small right‐shift at 10 μmol L^−1^ (Figure 5D).

### 3.5 Rat trachea

**Figure 6:**
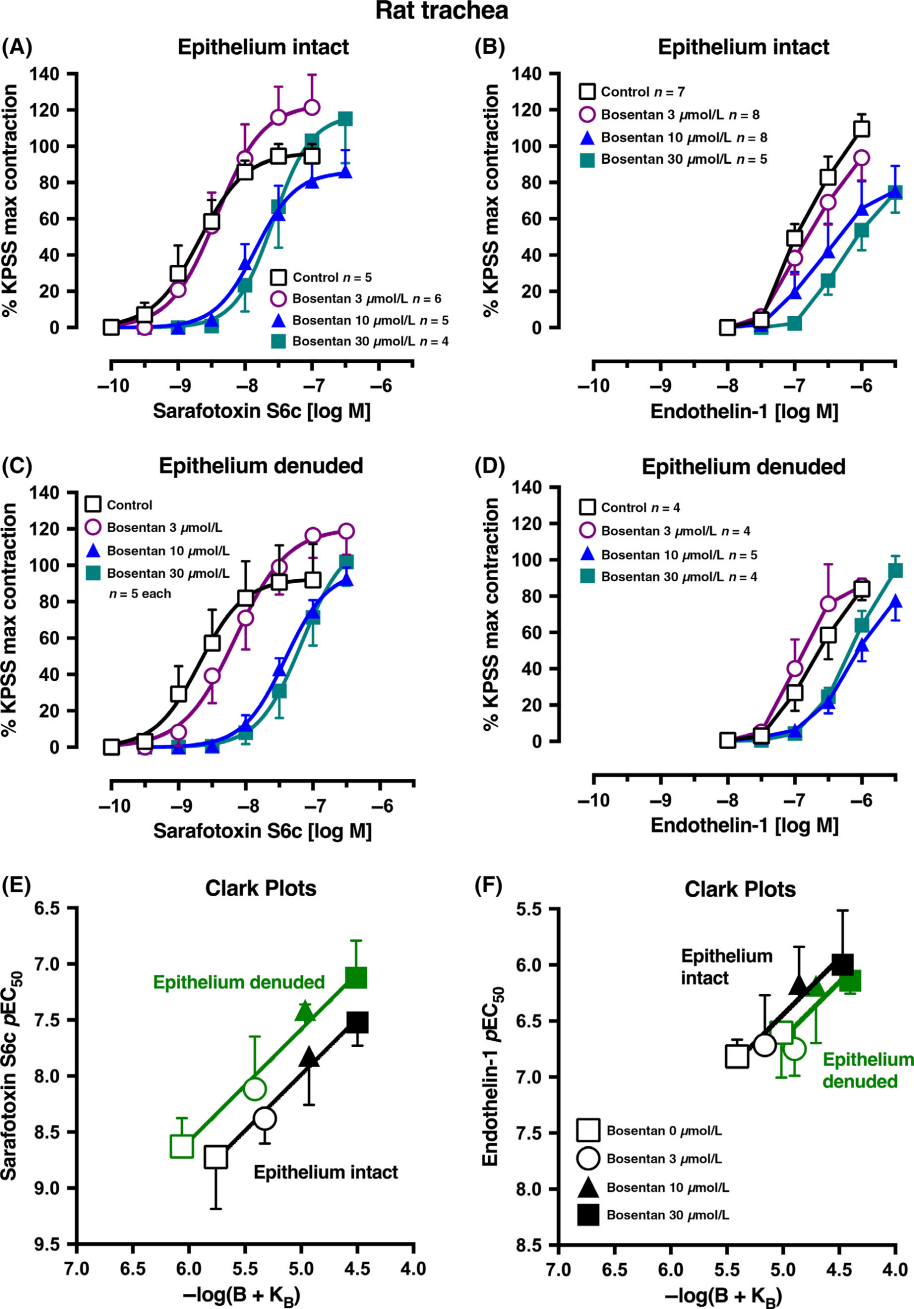
Average single concentration‐contraction curves to (A) sarafotoxin S6c or (B) endothelin‐1 in rat isolated trachea with intact epithelium in the absence (Control, 0 μmol L^−1^) or presence of bosentan 3, 10, or 30 μmol L^−1^. (C‐D) Corresponding agonist curves in trachea with epithelium denuded. Data are expressed as % KPSS maximum contraction (y axis). Error bars in (A‐D) are ± 1 SEM (those not shown are contained within the symbol). (E‐F) Clark plot displays for the relationship in the rat trachea between the (E) sarafotoxin S6c or (F) endothelin‐1 *p*EC_50_ values (y axis; −log M) and −log(B + K_B_) where B is concentration of bosentan (0, 3, 10, or 30 μmol L^−1^) and K_B_ is the global‐fitted dissociation constant (see legend for Figure 1C). n, number of tracheal rings isolated from separate animals. Variations in *n* are due to violation of a predetermined criterion: tracheae that contracted to KPSS with <1 g force

As was observed in rat small pulmonary artery, sarafotoxin S6c was significantly more potent than endothelin‐1 in contracting the rat isolated trachea with the epithelium intact in the absence of any antagonist (*p*EC_50_ values: sarafotoxin S6c 8.72 ± 0.22, n* *=* *4; endothelin‐1 6.82 ± 0.09, n* *=* *7; Figure 6A,B). In the absence of epithelium, the *p*EC_50_ for sarafotoxin S6c was not changed (8.63 ± 0.13, n* *=* *5) and similarly for endothelin‐1 (6.61 ± 0.17, n* *=* *4) (Figure 6C,D). The low potency of endothelin‐1 in rat trachea prevented exploration of the full concentration‐response curve, and therefore, we calculated *p*EC_50_ values at responses of 50% KPSS maximum contraction. In contrast, the full sarafotoxin S6c concentration‐response curves were obtained to allow *p*EC_50_ values to be calculated from logistic curve analysis, often when the Emax was more than 100% KPSS.

Bosentan (3‐30 μmol L^−1^) right‐shifted the endothelin‐1 and sarafotoxin S6c concentration‐contraction curves. The Clark plot and analysis show that with sarafotoxin S6c and epithelium intact, bosentan's *p*K_B_ was 5.76 ± 0.23 (n* *=* *20 points), not significantly different from endothelin‐1 as agonist with a *p*K_B_ of 5.41 ± 0.28 (n* *=* *28 points; Figure 6E,F). In epithelium‐denuded trachea, with sarafotoxin S6c, the bosentan *p*K_B_ was 6.06 ± 0.18 (n* *=* *20 points), significantly higher than for endothelin‐1 as agonist (*p*K_B_ 5.02 ± 0.31, n* *=* *17 points).

### 3.6 Modeling

To model the interaction between endothelin‐1 clearance and ET_B_ and ET_A_ receptor antagonism of the contraction response in small pulmonary arteries, we set the following criteria:

1. The ET_B_ receptor‐sensitive endothelin‐1 clearance mechanism (C_ETB_) can decrease the endothelin‐1 concentration at the ET_A_ or ET_B_ receptor environment by a maximum of 10‐fold (1 *p*EC_50_ unit).
2. The theoretical dual ET_A_ and ET_B_ receptor antagonist has the same *p*K_B_ value (8.5) at the “clearance ET_B_ receptor” as at the ET_B_ and ET_A_ receptor modulating contraction.
3. The efficiency of endothelin‐1 at ET_A_ and ET_B_ constrictor receptors is the same.

**Figure 7:**
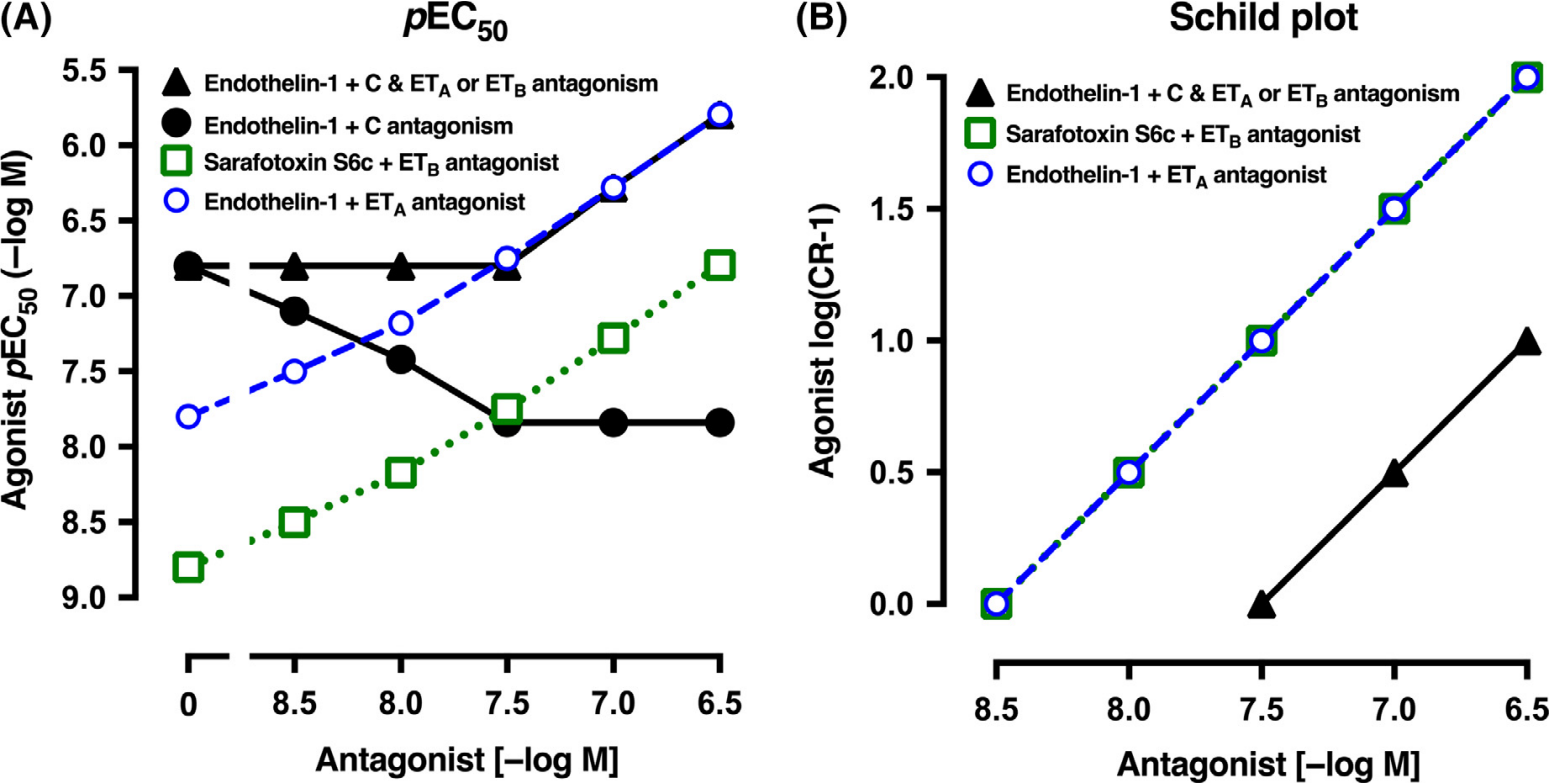
(A) Hypothetical relationship between the *p*EC_50_ values for endothelin‐1 or sarafotoxin S6c concentration‐contraction curves and the concentration of a theoretical dual ET_A_ and ET_B_ receptor antagonist with a *p*K_B_ of 8.5 at both receptors. For simplicity, the control (0 antagonist) *p*EC_50_ for sarafotoxin S6c (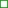) was set 1 log unit higher (10‐fold more potent) than for endothelin‐1 (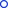). In the presence of ET_B_ receptors and the clearance (C) mechanism for endothelin‐1, the maximum clearance was set at 10‐fold (1 log unit) so that the *p*EC_50_ in the presence of no antagonist (0) rises 1 log unit (● or ▲). As the ET_B_ antagonism starts to block the endothelin‐1 clearance, so the *p*EC_50_ rises (●) but just as does the ET_B_ and ET_A_ antagonism so that the resultant shows the actual *p*EC_50_ is not altered. (B) The Schild plot for endothelin‐1 (or sarafotoxin S6c) as the agonist and the dual ET_A_ and ET_B_ antagonist with *p*K_B_ of 8.5 is shown. Separate theoretical lines are shown for ET_A_ (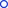; eg, rat aorta) and ET_B_ (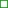; eg, trachea). In the presence of ET_B_‐mediated clearance (C) that removes endothelin‐1, as in pulmonary artery, the Schild plot points (▲) move parallel 1 log unit to decrease the potency of the dual antagonist by 10‐fold (ie, the *p*K_B_ of 8.5 becomes 7.5). The y axis is the agonist log(concentration ratio–1) and the x axis shows the concentration of dual ET_A_ and ET_B_ antagonist (−log M)

In Figure 7A, we set the control *p*EC_50_ for the sarafotoxin S6c concentration‐response curve at 8.8 so that a twofold shift (log 0.3) would result in a *p*EC_50_ of 8.5 in the presence of an ET_A_ and ET_B_ antagonist with a *p*K_B_ of 8.5 (3 nmol L^−1^). Similarly, we set the control *p*EC_50_ for endothelin‐1 at 7.8. Assuming that the ET_B_ (sarafotoxin S6c) assay was not compromised by clearance nor the endothelin‐1 assay (like mesenteric artery or rat aorta), then the *p*EC_50_ in the presence of the dual ET receptor antagonist (8.5‐6.5 −log M) would rise as shown in Figure 7A and the Schild plot would show competitive antagonism (slope = 1) and *p*K_B_ 8.5 (Figure 7B). If the assay is of predominantly ET_B_ receptors and the clearance mechanism is active, as we hypothesize in the small pulmonary artery, the control *p*EC_50_ for endothelin‐1 would lower to 6.8 as endothelin‐1 is removed from the receptor locus by the clearance mechanism (Figure 7A; ●). In the presence of the dual ET_A_ and ET_B_ antagonist, endothelin‐1 would be both potentiated in concentration available to ET_B_ receptors as the clearance mechanism is antagonized, but in addition, the endothelin‐1 concentration would be inhibited at the ET_B_ receptor mediating contraction. This is shown graphically in Figure 7A where at 3 concentrations of the dual antagonist, the endothelin‐1 *p*EC_50_ is both enhanced (●) and antagonized (▲) with 8.5, 8, 7.5, 7, and 6.5 −log M. To the experimenter of course, only the resultant of the potentiation and antagonism might be observed as shown by the ▲ flat line until surmountable antagonism is observed at 7 (–log M).

The relationship between the endothelin‐1 *p*EC_50_ and the ET_A_ and ET_B_ receptor antagonist concentrations is best illustrated in a Schild plot (Figure 7B). The dual ET_A_ and ET_B_ antagonist in the absence of the ET_B_ clearance mechanism shows slope 1 and *p*K_B_ 8.5 for the agonists endothelin‐1 at an ET_A_ receptor‐only assay and *p*K_B_ 8.5 for sarafotoxin S6c at an ET_B_ receptor assay. Note that at *p*Antagonist 6.5, the ET_A_ and ET_B_ shift is 100‐fold (log(concentration ratio − 1) = 2). But if the endothelin‐1 clearance mechanism is active, as in small pulmonary arteries, the shift at constrictor ET_B_ receptors is now only 10‐fold at *p*Antagonist 6.5 as the *p*K_B_ has moved to 7.5. Clinically, this scenario would demand at least a plasma level of endothelin antagonist of *p*6.5 (ie, 0.3 μmol L^−1^) if the *p*K_B_ at ET_A_ and ET_B_ receptors was 8.5.

**Figure 8:**
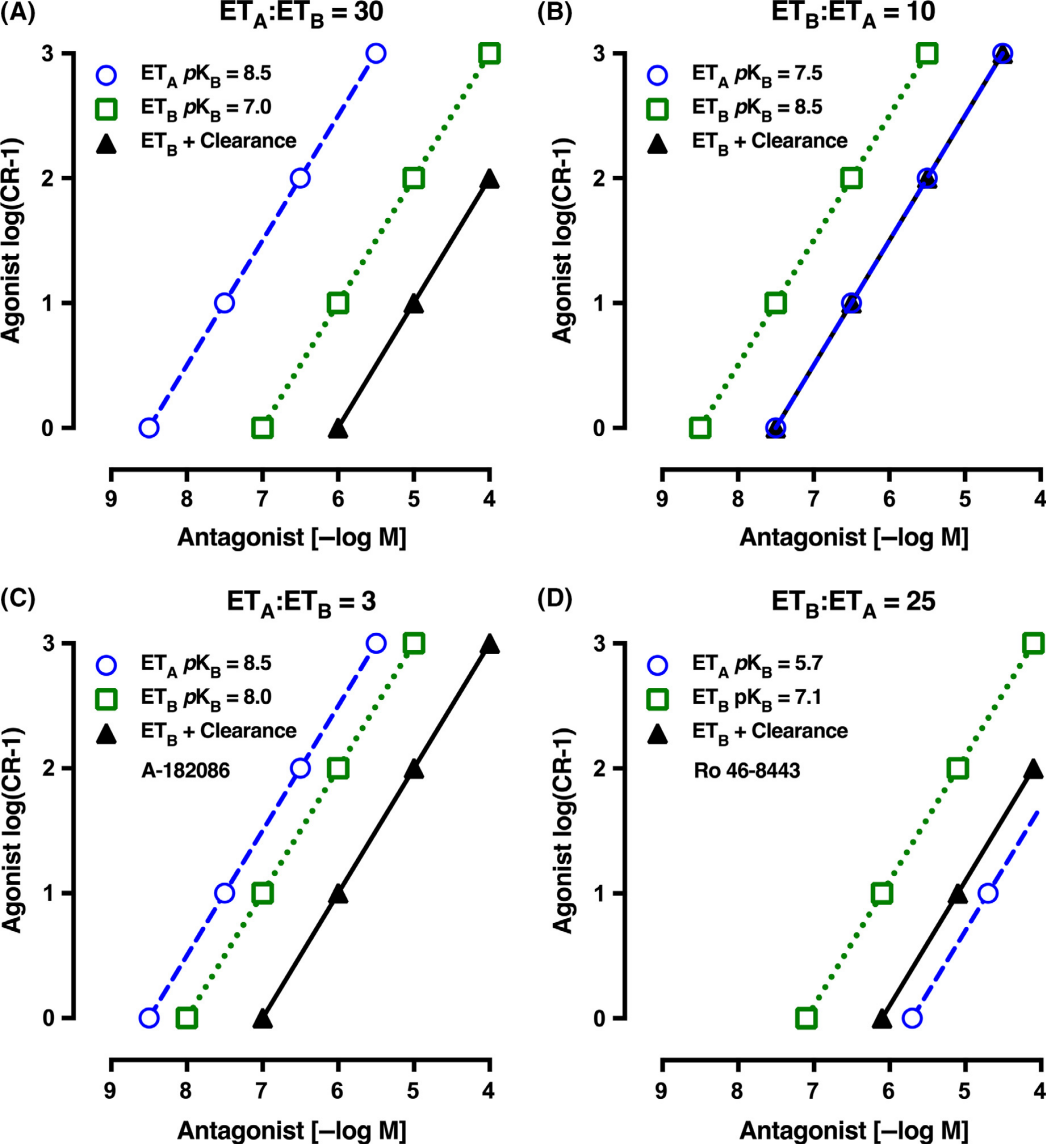
(A) Schild plot for a theoretical endothelin‐1 antagonist that is 30‐fold more selective at ET_A_ vs ET_B_ receptors (*p*K_B_: ET_A_ 8.5 and ET_B_ 7.0). Note that in the presence of ET_B_‐mediated clearance, the plasma concentration of the dual antagonist must rise to 10 μmol L^−1^ to give a 10‐fold antagonism at ET_B_ constrictor receptors and 3000‐fold antagonism at ET_A_ constrictor receptors. (B) Schild plot for a theoretical endothelin‐1 antagonist that is 10‐fold more selective at ET_B_ vs ET_A_ receptors (*p*K_B_: ET_B_ 8.5 and ET_A_ 7.5). In the presence of ET_B_‐mediated clearance, the plasma concentration of the dual antagonist must rise to 0.3 μmol L^−1^ to give a 10‐fold antagonism at both ET_B_ and ET_A_ receptors. (C) Schild plot for endothelin‐1 antagonist A‐182086 that is threefold more selective for ET_A_ vs ET_B_ receptors (*p*K_B_: ET_A_ 8.5 and ET_B_ 8.0; see Table 1). In the presence of ET_B_‐mediated clearance, the plasma concentration of the dual antagonist must rise to 1 μmol L^−1^ to give a 10‐fold antagonism at ET_B_ constrictor receptors and 300‐fold antagonism at ET_A_ constrictor receptors. (D) Schild plot for endothelin‐1 antagonist Ro 46‐8443 that is 25‐fold more selective at ET_B_ vs ET_A_ receptors (*p*K_B_: ET_B_ 7.1 and ET_A_ 5.7; see Table 1). In the presence of ET_B_‐mediated clearance, the plasma concentration of the dual antagonist must rise to 7.9 μmol L^−1^ to give a 10‐fold antagonism at ET_B_ receptors, with a fivefold antagonism at ET_A_ receptors. The y axis is the agonist log(concentration ratio–1) and the x axis shows the concentration of dual ET_A_ and ET_B_ antagonist (−log M)

Two further scenarios have been modeled. First, if the ET_A_ to ET_B_ selectivity ratio was ET_A_‐selective by 30‐fold, ie, *p*K_B_ for the ET antagonist at ET_A_ receptors was 8.5 and 7.0 for ET_B_ receptors, the Schild plot with endothelin‐1 clearance active shows that to reach a 10‐fold antagonism at ET_B_ constrictor receptors, then the plasma concentration would need to reach 10 μmol L^−1^ (*p*5 mol L^−1^), and at this concentration, the antagonism of ET_A_ receptors would be 3000‐fold (Figure 8A). The second scenario is when an endothelin antagonist has a 10‐fold selectivity for ET_B_ over ET_A_ receptors; thus, *p*K_B_ at ET_B_ receptors is set at 8.5 and for ET_A_ receptors at 7.5. The Schild plot (Figure 8B) shows that endothelin‐1 clearance will effectively decrease the *p*K_B_ of antagonism at ET_B_ constrictor receptors to 7.5, the same as ET_A_. Thus, a concentration of 0.3 μmol L^−1^ would result in a 10‐fold shift in both ET_A_ and ET_B_ receptors in the presence of endothelin‐1 clearance.

We also present the Schild plot for compound A‐182086 which was developed with just threefold ET_A_ to ET_B_ selectivity (*p*K_B_ at ET_A_ 8.5 and at ET_B_ 8; Figure 8C). Thus, in the presence of clearance, the plasma levels would need to be about 1 μmol L^−1^ (ie, 6 −log M) for a 10‐fold shift in ET_B_ receptor constrictor activity, while there would be at least 300‐fold shift for ET_A_ receptors. Indeed, these peak plasma levels of 4.3 μmol L^−1^ were achieved in rats given A‐182086 10 mg·kg^−1^ oral or even greater in dogs (34.5 μmol L^−1^), but significantly less in monkeys (0.16 μmol L^−1^) as the bioavailability varied from 54%, 71%, and 11%, respectively.^24^

Finally, we present the theoretical Schild plot for a selective ET_B_ antagonist Ro 46‐8443 where the *p*K_B_ at ET_B_ is 7.1 and 5.7 at ET_A_ receptors (Figure 8D). This ET_B_ to ET_A_ selectivity of 25 shows an important effect that when even with clearance in operation there is still more ET_B_ constrictor antagonism than ET_A_.

## 4 DISCUSSION

Our work supports the hypothesis that in very specific vascular beds, the local clearance of endothelin‐1 lowers the endothelin‐1 concentration that would activate ET_A_ or ET_B_ endothelin receptors. The *p*K_B_ estimate for ET_A_, ET_B_, or mixed ET_A_ and ET_B_ receptor antagonists will be confounded by 2 competing processes: one to potentiate the agonist endothelin‐1 and the second to antagonize its action at ET_A_ and/or ET_B_ receptors.

The tissue assays reported here confirm that there are special defined locations in some vascular beds and tracheal tissue that have a major population of ET_B_ receptors on smooth muscle. Functional ET_B_ receptors were defined by the substantial contraction up to the tissue maximum by the potent ET_B_‐selective agonist sarafotoxin S6c. This agonist is not a substrate for the ET_B_ receptor‐sensitive clearance mechanism specifically shown for endothelin‐1 and blocked by ET_B_ antagonists. Thus, the rat tracheal ring with agonist sarafotoxin S6c and epithelium intact proved to be a robust assay to define the *p*K_B_ for ET_B_ antagonists. We calculated the *p*K_B_ for bosentan as 5.76 ± 0.23 for ET_B_ receptors with sarafotoxin S6c and similarly 5.41 ± 0.28 with endothelin‐1. Importantly, the *p*K_B_ for bosentan and sarafotoxin S6c was the same whether the epithelium was present or absent (*p*K_B_ 5.76 ± 0.23 and 6.06 ± 0.18, respectively). In the original bosentan report, in rat tracheal rings, the *p*A_2_ was reported as 5.94 ± 0.04 with Schild slope 0.90 ± 0.18.^17^ Thus, tracheal smooth muscle ET_B_ receptors mediate contraction, but there is no evidence of clearance of endothelin‐1 in this assay.

For the ET_A_ receptor, the analysis is less certain as there is no selective ET_A_ receptor agonist.^25^ The main assay used to determine the *p*K_B_ (7.28) for bosentan at ET_A_ receptors was the contraction to endothelin‐1 of rat aortic rings, with endothelium removed.^26^ Our competitive *p*K_B_ values for bosentan and endothelin‐1 in human large diameter arteries such as pulmonary (i.d. 5.5 mm) and radial (i.d. 3.23 mm) 27 and in rat mesenteric small artery (i.d. 0.25 mm) or mouse main pulmonary (i.d. 0.65 mm), mesenteric (i.d. 0.28 mm), and tail (i.d. 0.37 mm) arteries all fall in the range 6.04‐7.31, consistent with ET_A_ receptor antagonism. The one outstanding artery, of those we tested, where the dual ET_A_ and ET_B_ antagonist bosentan was apparently very weak was the rat small pulmonary artery.

There are 3 possible factors that could affect the *p*K_B_ estimation: (i) endothelin‐1 could activate endothelial ET_B_ receptors to release nitric oxide to functionally antagonize the contraction through smooth muscle cell ET_A_ or ET_B_ receptors; (ii) in some arteries, there may be a mix of ET_A_ and ET_B_ receptors; and (iii) the ET_B_ receptor‐mediated clearance mechanism has an important action to decrease endothelin‐1 local concentrations by as much as 10‐fold.

First to the role of nitric oxide, L‐NAME made no significant difference to the *p*K_B_ estimation for bosentan in rat small pulmonary artery (Figure 2). L‐NAME (100 μmol L^−1^) was effective in eliminating the release of NO as demonstrated by the complete abolition of the relaxation to acetylcholine (1 μmol L^−1^) in U46619‐precontracted arteries. Second, despite L‐NAME being present, endothelin‐1 was much less potent (lower *p*EC_50_) in rat pulmonary small artery than in rat mesenteric artery. Third, in the presence of bosentan 10 μmol L^−1^, the *p*EC_50_ for endothelin‐1 was right‐shifted and lowered to 7.2 (−log M) in the mesenteric artery, while in contrast, it was left‐shifted and raised to a *p*EC_50_ of 8.7 compared with control in the pulmonary artery (Figure 1A,B). We suggest that this anomalous result and inability to determine a *p*K_B_ with bosentan in rat pulmonary artery is explained by the continuous removal of endothelin‐1 by the ET_B_ receptor‐sensitive clearance mechanism found in this particular artery, but not in the rat mesenteric artery or aorta, nor human large pulmonary or radial artery.^27^

**Figure 9:**
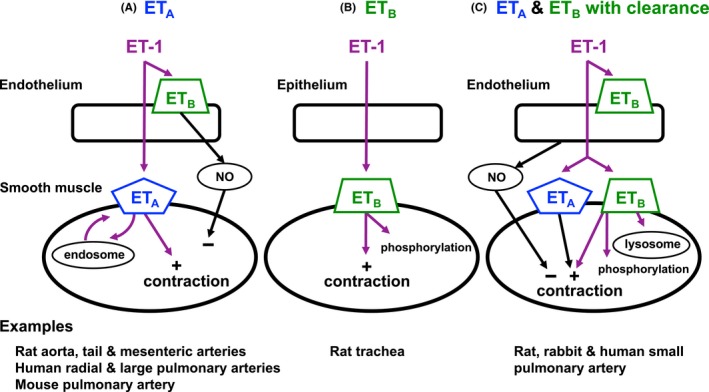
Schematic diagram of the location and function of ET_A_ and ET_B_ receptors in 3 tissue assays. (A) ET_A_ receptors located on vascular smooth muscle mediate contraction. ET_A_ receptors are internalized and recycled slowly through endosomes.^36^ (B) ET_B_ receptors located on smooth muscle cells mediate contraction and are rapidly removed by phosphorylation.^36^ (C) ET_B_ receptors located on smooth muscle cells bind endothelin‐1 and clear endothelin‐1 from the environment through lysosomal metabolism. The remaining endothelin‐1 binds to ET_A_ and ET_B_ receptors on smooth muscle to mediate contraction before being recycled by endosomes or destroyed by phosphorylation, respectively. In (A) and (C), ET_B_ receptors on the endothelium mediate release of NO that transiently relaxes smooth muscle. Examples of the species and tissues assumed to have these particular receptor profiles are given below each panel. ET‐1, endothelin‐1. NO, nitric oxide

Further, direct functional evidence of the clearance of endothelin‐1 in rat pulmonary artery comes from the selective ET_B_ antagonist BQ788 assay. With the agonist sarafotoxin S6c, and L‐NAME present, the pattern of BQ788 competitive antagonism shows right‐shifted concentration‐response curves with a *p*K_B_ of 7.2 ± 0.21 from Clark plot analysis (Figure 3D,H). In stark contrast, when endothelin‐1 was the agonist, BQ788 up to 3 μmol L^−1^ caused no significant rightward shift, if anything a small left‐shift indicative of blockade of endothelin‐1 ET_B_ clearance (Figure 3B,F). Removal of the endothelium failed to change the pattern of the endothelin‐1 and BQ788 interaction (Figure 3A,E). But bosentan was close to being a competitive ET_A_ antagonist in the pulmonary artery in the presence of L‐NAME *AND* BQ788 to antagonize the clearance of endothelin‐1 (Figure 3C,G). Similarly, in human pulmonary resistance arteries, the ET_A_ receptor antagonist BMS182874 was ineffective against low concentrations of endothelin‐1.^28^ The finding that endothelium removal did not affect the EC_50_ nor Emax to endothelin‐1 or change the action of BQ788 in the rat small pulmonary artery compared to endothelium‐intact tissues suggests that the arterial smooth muscle cells are the primary location of the proposed clearance mechanism (Figure 9).

In earlier work, Hay et al^29^ reported that in rabbit pulmonary artery, sarafotoxin S6c gave a *p*K_B_ of 7.7 for the mixed ET_A_ and ET_B_ receptor antagonist SB209670, but 6.7 when endothelin‐1 was the agonist. In human small pulmonary arteries (150‐200 μm i.d.) sarafotoxin S6c was more than 100‐fold more potent than endothelin‐1 and the authors concluded that both ET_A_ and ET_B_ receptor antagonists are required to antagonize endothelin‐1.^28^ We also found that the apparently weak antagonism of endothelin‐1 by bosentan in rat pulmonary arteries is shared with ambrisentan and macitentan (Figure 5). Indeed, these latter 2 endothelin‐1 antagonists are more ET_A_ than ET_B_ receptor selective (Table 1). However, it is important to note that the active metabolite of macitentan ACT‐132577 may additionally play a significant role as a dual ET_A_/ET_B_ antagonist in vivo. Other factors such as pharmacokinetic differences will also affect the translation of these isolated tissue assay results into the clinic. Thus, there are a number of species including man, rabbit, and rat where small interlobar pulmonary arteries potentially have the ET_B_‐sensitive endothelin‐1 clearance mechanism and significant populations of ET_B_ and ET_A_ receptors on the smooth muscle mediating contraction.

**Table 1:** Estimates of *p*K_B_ from functional isolated tissue assays and selectivity ratios for dual ET_A_ and ET_B_ receptor antagonists in the absence or presence of endothelin‐1 clearance

	Receptor assay *p*K_B_			Ratio	
Endothelin antagonist	ET_A_ Aorta^g^^,^ h	ET_B_ Trachea^g^^,^ i	ET_B_ + C^j^	ET_A_:ET_B_ k	ET_A_:ET_B_ + C^l^
Bosentan^a^	7.3	5.9	4.9	25	250
Ambrisentan^b^	7.1	5.6	4.6	32	320
Macitentan^c^	7.6	5.9	4.9	50	500
A‐182086^d^	8.5^m^	8.0^m^	7.0	3	30
Ro 46‐8443^e^	5.7	7.1	6.1	0.04	0.4
“Ideal”^f^	8.5	8.5	7.5	1	10

a Clozel et al.^17^

b Bolli et al.^33^

c Iglarz et al.^34^

d Wessale et al.^24^

e Breu et al.^35^

f “Theoretical dualist.”

g *p*K_B_ from Schild plots.

h Rat aorta (without endothelium) and agonist endothelin‐1.

i Rat trachea (without epithelium) and agonist sarafotoxin S6c.

j *p*K_B_ for ET_B_ receptors under the influence of endothelin‐1 clearance theoretically taken to be 10‐fold (ie, 1 log unit).

k ET_A_ to ET_B_ selectivity ratio calculated as antilog (*p*K_B_ ET_A_ − *p*K_B_ ET_B_).

l ET_A_ to ET_B_ + C selectivity ratio calculated as antilog (*p*K_B_ ET_A_ − *p*K_B_ ET_B_ + C).

m With endothelium.

Rabbit pulmonary artery (without endothelium).

### 4.1 Potential clinical implications

We have analyzed the endothelin receptor pharmacology in a wide range of arteries and the small pulmonary artery appears to be unique with its mix of ET_A_ and ET_B_ receptors and clearance mechanism. If this finding can be extrapolated to the clinic, there are a number of caveats that must be considered. (i) There is evidence in rats with monocrotaline‐induced pulmonary hypertension that the ET_B_ receptor mRNA and protein expression in small pulmonary arteries are down‐regulated.^30^ However, in patients with severe pulmonary artery hypertension, the ET_B_ receptor mRNA and protein expression were upregulated in the media of pulmonary arteries, while the ET_A_ receptor gene expression was unaffected.^31^ (ii) That ET_B_ receptors on endothelial cells are protective in limiting vascular remodeling and development of pulmonary hypertension, so that ET_B_ antagonism may well be deleterious.^32^ (iii) That pharmacokinetic actions and active metabolites together with protein binding will significantly alter the resultant activity of endothelin receptor antagonists.

Noting the above, there are 3 dual endothelin antagonists approved for pulmonary artery hypertension. On isolated tissue assay data, all have a significant ET_A_ to ET_B_ receptor selectivity ratio of 25‐50 (Table 1). Our theoretical modeling which reflects our in vitro experimental data (Figures 1 and 8A) suggests that to obtain a 10‐fold antagonism of the ET_B_ constrictor receptor in the presence of clearance, then a plasma concentration of 3000 times higher than the *p*K_B_ at ET_A_ receptors would be required. For bosentan, for example, plasma levels would need to be in the range of 200 μmol L^−1^! If these levels are not obtained, the antagonist would generally behave only as an effective ET_A_ antagonist in the clinic.

Our modeling suggests that a 10‐fold selective ET_B_ vs ET_A_ antagonist might be ideal in antagonizing the pulmonary artery ET_B_ receptors in the presence of CLEARANCE (Figure 8B). Ro 46‐8443^35^ is 25‐fold selective for ET_B_ vs ET_A_, and modeling would suggest that with a *p*K_B_ of 7.1 at ET_B_ receptors (Figure 8D), a plasma level would be required of nearly 10 μmol L^−1^ to give a 10‐fold antagonism of ET_B_ receptors, but ET_A_ antagonism would not be sufficient if inhibition of clearance presented a higher level of endothelin‐1. Another nonselective and potent ET_A_ and ET_B_ antagonist with selectivity ratio of just 3, A‐182086 (Table 1), has been used in animals and shows that effective ET_A_ and ET_B_ receptor antagonism was achieved after oral dosing.^24^

Given that any antagonism of clearance will raise plasma endothelin‐1 levels, there must be sufficient ET_A_ receptor antagonism present to obviate vasoconstriction from this raised endothelin‐1 concentration. Theoretically then, an ET_A_ vs ET_B_ selectivity of 10‐fold would be sufficient, provided a high plasma concentration is achieved for ET_B_ antagonism. From Table 1, we predict that given clearance of endothelin‐1 in important tissues such as pulmonary artery, the effective ET_B_ antagonism is 10‐fold weaker so that the ET_A_ to ET_B_ + clearance selectivity ratio increases by 10‐fold. In effect, this suggests that the 3 antagonists in the clinic for pulmonary artery hypertension are principally ET_A_‐selective agents. The “ideal” antagonist would have identical *p*K_B_ values at ET_A_ and ET_B_ receptors.

## 5 CONCLUSION

This experimental work in isolated tissue assays offers an explanation for the mechanism of the failure of “dual” ET_A_ and ET_B_ antagonists to competitively antagonize endothelin‐1 in some important arteries such as the small pulmonary artery where ET_A_ and ET_B_ receptors predominate to cause contraction. The experimental results and theoretical modeling are consistent with an endothelin‐1 clearance mechanism through internalization of endothelin‐1 bound to ET_B_ receptors on smooth muscle of some blood vessels that can lower the endothelin‐1 concentration by 10‐fold. When this mechanism is blocked by ET_B_ antagonists, the endothelin‐1 concentration will rise. The combination of a possible endothelin‐1 clearance and contraction mediated by ET_A_ and ET_B_ receptors provides an environment that would prevent effective endothelin‐1 receptor antagonism. This conclusion may have important implications for the effective use of endothelin antagonists in the treatment of pulmonary artery hypertension.

## AUTHORS’ CONTRIBUTIONS

J.A.A. and C.E.W. conceived the study and designed the protocol. R.J.A.H. performed wire myography studies. J.A.A., C.E.W., and R.J.A.H. collected and analyzed data. J.A.A. and C.E.W. wrote the manuscript. R.J.A.H. critically reviewed the manuscript. All authors approved the final version of the manuscript.

## DISCLOSURES

None declared.
